# Phloretin Promotes Adipogenesis via Mitogen-Activated Protein Kinase Pathways in Mouse Marrow Stromal ST2 Cells

**DOI:** 10.3390/ijms19061772

**Published:** 2018-06-14

**Authors:** Ayumu Takeno, Ippei Kanazawa, Masakazu Notsu, Ken-ichiro Tanaka, Toshitsugu Sugimoto

**Affiliations:** Internal Medicine 1, Shimane University Faculty of Medicine, 89-1, Enya-cho, Izumo, Shimane 693-8501, Japan; atakeno@med.shimane-u.ac.jp (A.T.); mnotsu25@med.shimane-u.ac.jp (M.N.); ken1nai@med.shimane-u.ac.jp (K.T.); sugimoto@med.shimane-u.ac.jp (T.S.)

**Keywords:** phloretin, adipogenesis, glucose uptake, marrow stromal ST2 cells, mitogen-activated protein kinase, adenosine monophosphate-activated protein kinase

## Abstract

Phloretin, a glucose transporter (GLUT) inhibitor, has pleiotropic effects. The present study examined the effects of phloretin on the commitment of marrow stromal cells to adipocytes, using the mouse marrow stromal cell line ST2. Oil red O staining showed that treatment with phloretin 10–100 µM promoted lipid accumulation. Real-time PCR showed that phloretin significantly increased the expression of adipogenic markers, including PPARγ, C/EBPα, fatty acid synthase, fatty acid-binding protein 4, and adiponectin. Western blotting showed that phloretin inhibited ERK1/2 and JNK but activated p38 MAPK. Treatment with a MAPK/ERK kinase inhibitor and a JNK inhibitor enhanced adipogenesis, similar to phloretin. In contrast, a p38 MAPK inhibitor suppressed phloretin-induced adipogenesis. Although phloretin phosphorylated AMP-activated protein kinase (AMPK), co-incubation with an AMPK inhibitor did not block phloretin-induced adipogenesis. The 2-deoxyglucose colorimetric assay showed that phloretin and siRNA silencing of GLUT1 decreased glucose uptake. However, unlike phloretin treatment, GLUT1 silencing inhibited adipogenesis. In addition, phloretin enhanced adipogenesis in GLUT1 knocked-down cells. Taken together, phloretin induced adipogenesis of marrow stromal cells by inhibiting ERK1/2 and JNK and by activating p38 MAPK. The adipogenic effects of phloretin were independent of glucose uptake inhibition. Phloretin may affect energy metabolism by influencing adipogenesis and adiponectin expression.

## 1. Introduction

Adipose tissue is a crucial organ that regulates energy homeostasis. In addition to energy storage, adipose tissue functions as an endocrine organ. Adipocytes secrete adipokines, such as adiponectin (APN), leptin, and resistin, thereby regulating whole body metabolism [1]. Accumulation of visceral fat causes the dysregulation of adipokine secretions, leading to metabolic abnormalities including insulin resistance [1]. The adipocyte differentiation process is orchestrated by a network of transcriptional cascades, involving molecules such as the CCAAT/enhancer-binding protein (C/EBP) family members and peroxisome proliferator-activated receptor γ (PPARγ), which play central roles in adipocyte differentiation by regulating the expression of adipocyte-specific genes [2,3,4]. 

Phloretin (2′,4′,6′-trihydroxy-3-(4-hydroxyphenyl)-propiophenone) is a natural phenolic compound present in fruits, such as apples [5] and strawberries [6]. It has been reported that phloretin has pleiotropic effects, including anti-oxidative [7,8,9] and anti-inflammatory activities [10,11]. In addition, phloretin is a known inhibitor of glucose transporter (GLUT) [12] and is reported to suppress cancer growth by inhibiting glucose uptake through GLUT2 [13,14,15,16]. The effect of phloretin on adipogenesis has also been reported. In vitro studies have shown that treatment with phloretin increases the expression of PPARγ and C/EBPα and promotes adipocyte differentiation in mouse 3T3-L1 preadipocytes and porcine primary adipocytes, respectively [17,18]. On the other hand, Huang et al. reported that phloretin promoted lipolysis in fully differentiated mature 3T3-L1 cells [19]. These results indicate that phloretin may facilitate adipogenesis in preadipocytes and promote lipolysis in mature adipocytes. Several in vivo studies have shown that the administration of phloretin improves hyperglycemia, insulin resistance [20,21], and obesity [20] in diabetic rodent models. However, no studies have investigated the effects of phloretin on the commitment of multipotent marrow stromal cells (MSCs) to the adipocytes and its underlying mechanisms, including the contribution of glucose uptake inhibition.

Adenosine monophosphate-activated protein kinase (AMPK) is known as an intracellular energy sensor and plays crucial roles in the regulation of whole body energy metabolism [22]. AMPK senses energy shortage and suppresses energy consumption, i.e., lipogenesis, protein synthesis, and gluconeogenesis, while enhancing energy production, i.e., glucose uptake and fatty acid oxidation [22,23]. It has been reported that AMPK is a negative regulator of adipogenesis [24,25,26,27]. However, its precise mechanism remains to be elucidated. Furthermore, previous studies have shown that mitogen-activated protein kinase (MAPK) family, including extracellular signal-regulated kinase (ERK) 1/2, c-Jun N-terminal kinase (JNK) and p38 MAPK are associated with adipocyte differentiation [2,28]. We and other researchers have previously reported that phloretin promotes the phosphorylation of AMPK [19,29] and alters the phosphorylation status of MAPKs [11,18,29,30,31] in various cell types. However, it is unclear whether the effects of phloretin on adipogenesis are mediated by AMPK or MAPKs. 

In this study, we examined the effects of phloretin on adipocyte differentiation using the mouse MSC line ST2, and the involvement of the downstream signalling pathways linked to AMPK and MAPK. We also investigated whether the effects of phloretin were mediated by glucose uptake inhibition through GLUT.

## 2. Results

### 2.1. Effects of Phloretin on Adipocyte Differentiation in ST2 Cells

**Figure 1:**
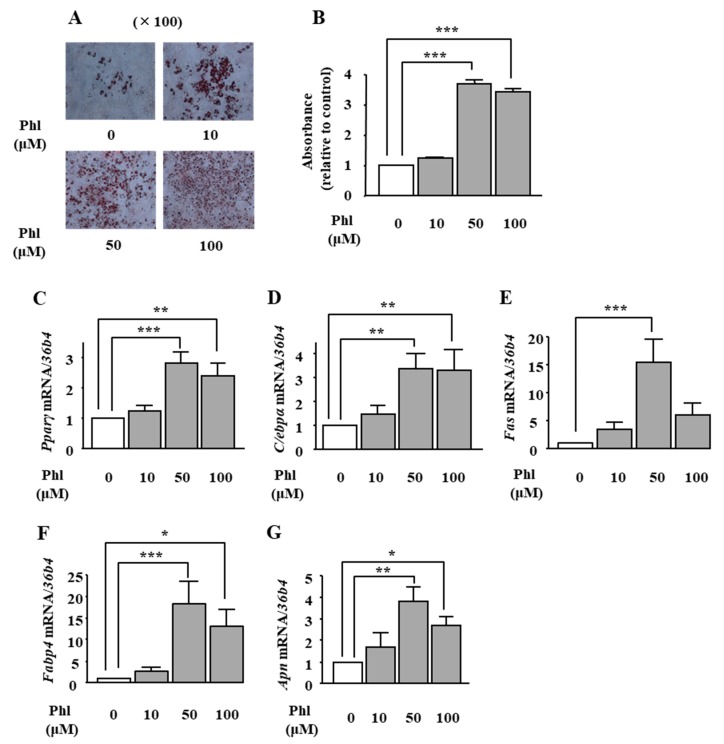
The effects of phloretin on adipocyte differentiation in ST2 cells. (**A**,**B**) After reaching confluency, ST2 cells were incubated in adipogenic medium with phloretin (0, 10, 50, and 100 µM), and oil red O staining was performed at day 8. The results are representative of at least seven different experiments. The quantification results are expressed as mean ± SE (*n* = 6); *** *p* < 0.001. (**C**–**G**) After reaching confluency, the cells were incubated in adipogenic medium. The mRNA expression of adipogenic differentiation markers (*Pparγ*, *C/ebpα*, *Fas*, *Fabp4*, and *Apn*) was examined by real-time PCR at day 4. The results are expressed as mean ± SE (*n* ≥ 5); * *p* < 0.05, ** *p* < 0.01, *** *p* < 0.001. Phl: phloretin.

We examined the effects of phloretin on adipogenesis in ST2 cells. Oil Red O staining and its quantification showed that treatment with phloretin (50 and 100 μM) significantly increased lipid deposition (Figure 1A), and the maximum effect was observed at 50 μM phloretin (Figure 1B). We also examined the mRNA expression of important adipogenic transcription factors (*Ppar*γ and *C/ebpα*) and adipocyte differentiation markers, fatty acid synthase (*Fas*), fatty acid-binding protein 4 (*Fabp4*) and adiponectin (*Apn*). Treatment with phloretin increased the mRNA expression of all the above-mentioned factors (Figure 1C–G). The maximum effects were observed at 50 μM phloretin (Figure 1C–G). the adipogenic markers *Pparγ*, *C/ebpα*. 

### 2.2. Role of AMPK in the Phloretin-Induced Upregulation of Adipocyte Differentiation Markers

**Figure 2:**
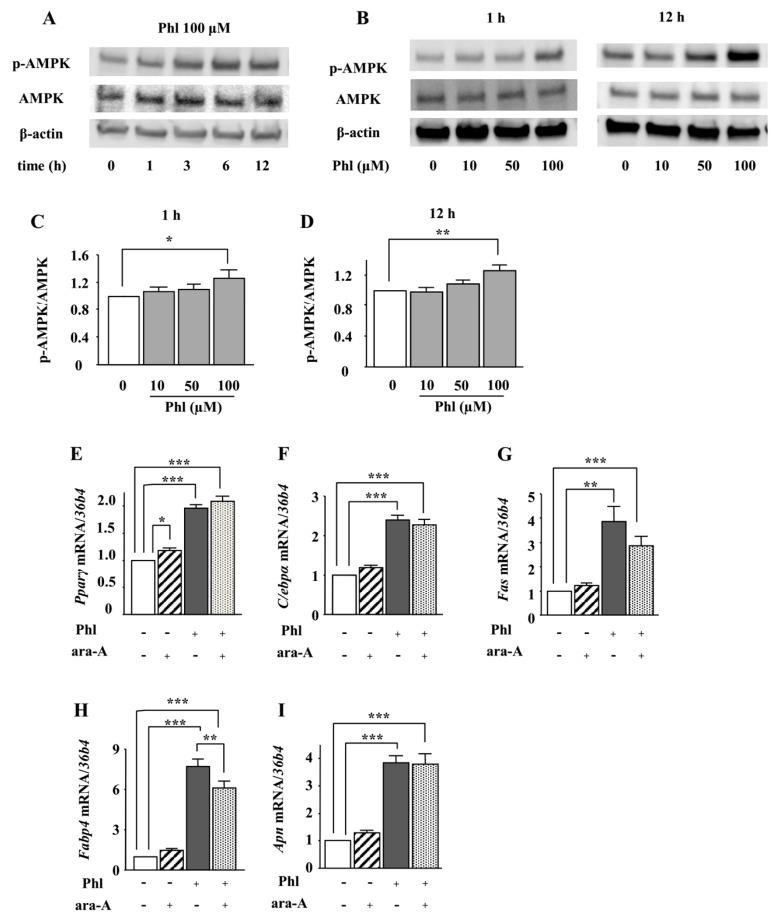
The effects of the AMPK inhibitor ara-A on phloretin-induced upregulation of adipocyte differentiation markers. (**A**–**D**) After reaching confluency, ST2 cells were incubated in adipogenic medium for 48 h. Thereafter, the cells were treated with 100 µM phloretin for up to 12 h, and western blot analysis was performed to examine the time-dependent effects of phloretin on AMPK (**A**). To test dose dependency, the cells were treated with phloretin (0 to 100 µM) for 1 and 12 h (**B**). Quantification of the bands was performed (**C**,**D**). The results are representative of at least four experiments. The quantification results are expressed as mean ± SE (*n* ≥ 4); * *p* < 0.05, ** *p* < 0.01. (**E**–**I**) After reaching confluency, the cells were incubated in adipogenic medium with 100 µM phloretin and/or 0.1 mM ara-A for 4 days. The mRNA expression of adipogenic differentiation markers (*Pparγ*, *C/ebpα*, *Fas*, *Fabp4*, and *Apn*) was examined by real-time PCR. The results are expressed as mean ± SE (*n* ≥ 7); * *p* < 0.05, ** *p* < 0.01, *** *p* < 0.001. Phl: phloretin.

Next, we examined whether AMPK is involved in phloretin-induced adipocyte differentiation in ST2 cells. After ST2 cells were incubated in adipogenic differentiation medium for two days, the effect of phloretin on the phosphorylation of AMPK was examined by western blotting. Treatment with phloretin (100 µM) enhanced the phosphorylation of AMPK (Figure 2A). Moreover, treatment with phloretin (10–100 µM) for 1 and 12 h dose-dependently enhanced the phosphorylation of AMPK (Figure 2B). The quantification of the bands showed that the increase in the ratio of phosphorylated AMPK to total AMPK was significant (Figure 2C,D). The treatment with the AMPK inhibitor ara-A (0.1 mM) alone did not alter the expression of the adipocyte differentiation markers (Figure 2F–I), although it slightly increased *Pparγ* expression (Figure 2E). Co-incubation with ara-A slightly but significantly suppressed phloretin-induced upregulation of *Fabp4* (Figure 2H), whereas the expression of other adipocyte differentiation markers was not affected (Figure 2E–G,I). These findings indicate that the phosphorylation of AMPK may not be associated with phloretin-induced upregulation of adipocyte differentiation markers in ST2 cells.

### 2.3. The Effects of Phloretin on the Phosphorylation of MAPKs in ST2 Cells

**Figure 3:**
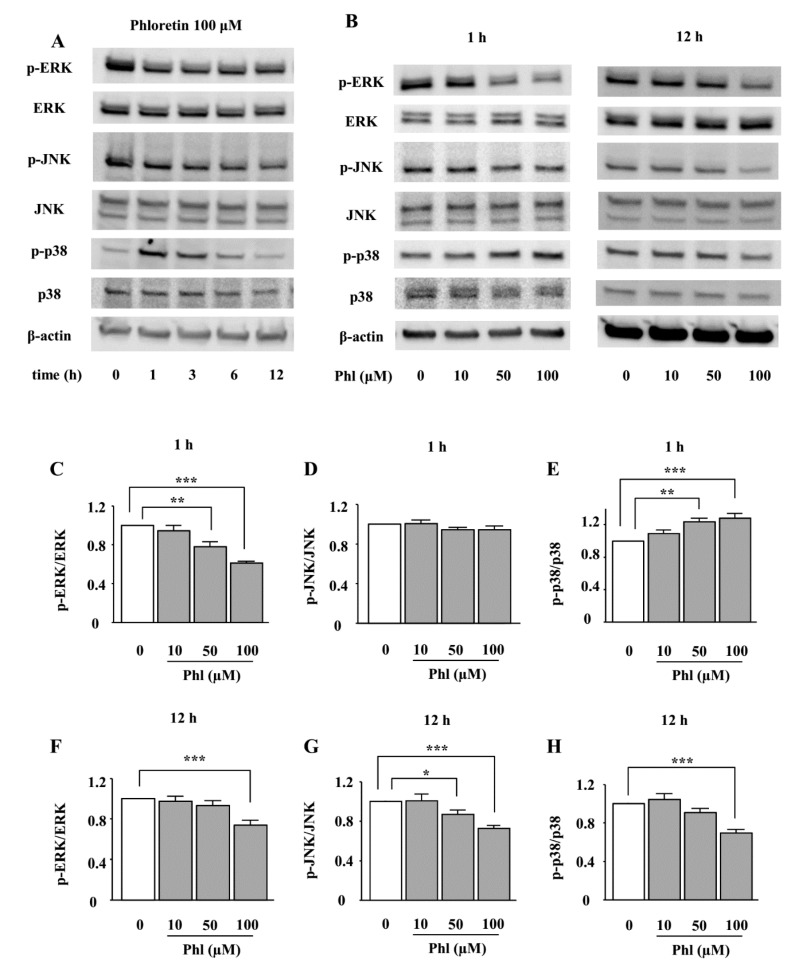
The effects of phloretin on the phosphorylation of MAPKs in ST2 cells. (**A**–**H**) After reaching confluency, ST2 cells were incubated in adipogenic medium for 48 h. Thereafter, the cells were treated with 100 µM phloretin for up to 12 h, and western blot analysis was performed to examine the time-dependent effects of phloretin on the phosphorylation of ERK1/2, JNK, and p38 MAPK (**A**). To test dose dependency, the cells were treated with phloretin (0 to 100 µM) for 1 and 12 h (**B**). The results are representative of at least five experiments. (**C**–**H**) The quantification results are expressed as mean ± SE (*n* ≥ 5); * *p* < 0.05, ** *p* < 0.01, *** *p* < 0.001. Phl: phloretin.

We examined the effects of phloretin on the phosphorylation of MAPKs, i.e., ERK1/2, JNK, and p38 MAPK. ST2 cells were incubated in adipogenic differentiation medium for two days, and then the effect of phloretin on the phosphorylation of MAPKs was examined by western blotting. The treatment with phloretin (100 µM) suppressed the phosphorylation of ERK1/2 and JNK up to 12 h (Figure 3A). Moreover, phloretin dose-dependently decreased the phosphorylation of ERK1/2 and JNK (Figure 3B). The densitometric analysis of the bands showed a significant decrease in the level of phosphorylated ERK1/2 at both 1 and 12 h, and of phosphorylated JNK at 12 h (Figure 3C,D,F,G). On the other hand, the treatment with phloretin (100 µM) transiently phosphorylated p38 MAPK, and, then, suppressed p38 MAPK expression (Figure 3A). Phloretin (10–100 µM) dose-dependently increased the phosphorylation of p38 MAPK at 1 h and decreased it at 12 h (Figure 3B). The densitometric analysis showed that the effects of phloretin on the phosphorylation of p38 MAPK were significant at both 1 and 12 h (Figure 3E,H).

### 2.4. The Effects of ERK1/2 Inhibition on Adipocyte Differentiation in ST2 Cells

**Figure 4:**
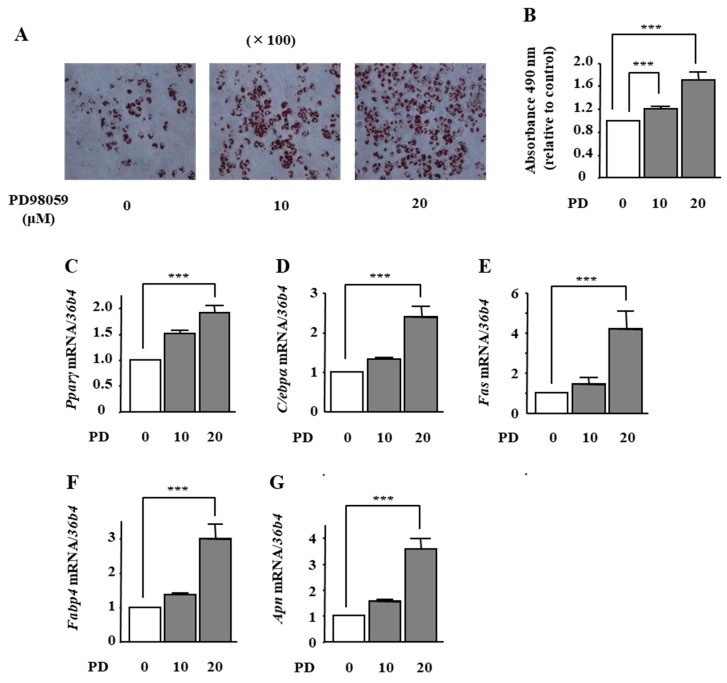
The effects of the MEK inhibitor PD98059 on adipocyte differentiation in ST2 cells. (**A**,**B**) After reaching confluency, ST2 cells were incubated in adipogenic medium with the MEK inhibitor PD98059 (0, 10, and 20 µM) for 8 days. Thereafter, oil red O staining and its quantification were performed. The results are representative of at least seven different experiments. The quantification results are expressed as mean ± SE (*n* = 8); *** *p* < 0.001. (**C**–**G**) After reaching confluency, the cells were incubated in adipogenic medium with PD98059 (0, 10, and 20 µM) for 4 days. The mRNA expression of adipogenic differentiation markers (*Pparγ*, *C/ebpα*, *Fas*, *Fabp4*, and *Apn*) was examined by real-time PCR. The results are expressed as mean ± SE (*n* = 7); *** *p* < 0.001. PD: PD98059.

To examine whether ERK1/2 inhibition is involved in phloretin-induced adipocyte differentiation in ST2 cells, we used the MAPK/ERK kinase (MEK) inhibitor PD98059. Treatment with PD98059 (10 and 20 μM) dose-dependently promoted lipid accumulation, as confirmed by oil red O staining (Figure 4A). The quantification of oil red O staining showed that the PD98059-induced increase in lipid deposition was significant (Figure 4B). In addition, the mRNA expression of *Pparγ*, *C/ebpα*, *Fas*, *Fabp4*, and *Apn* was significantly increased by PD98059 treatment (20 μM) (Figure 4C–G). 

### 2.5. The Effects of JNK Inhibition on Adipocyte Differentiation in ST2 Cells

**Figure 5:**
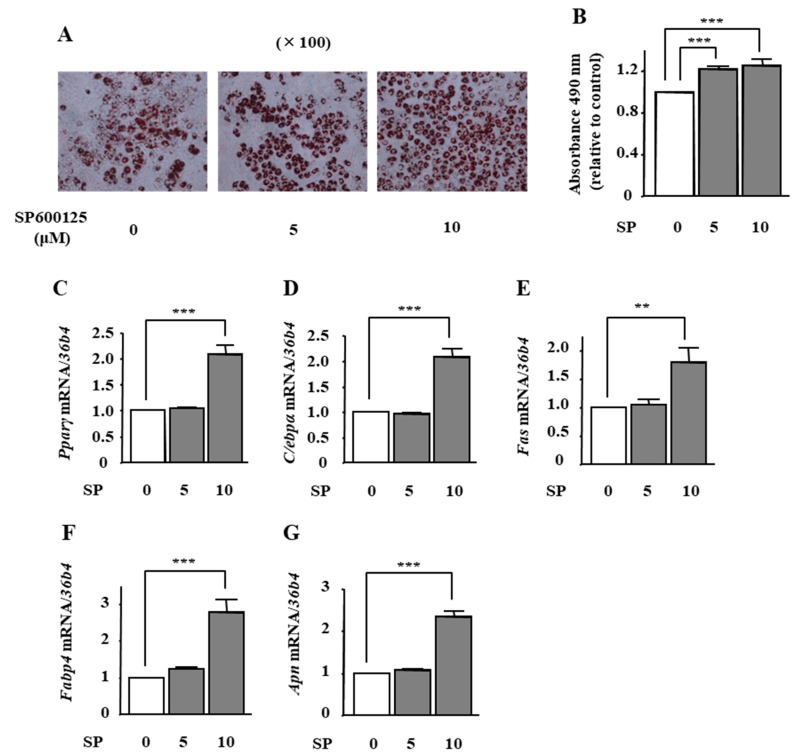
The effects of the JNK inhibitor SP600125 on adipocyte differentiation in ST2 cells. (**A**,**B**) After reaching confluency, ST2 cells were incubated in adipogenic medium with the JNK inhibitor SP6001215 (0, 5, and 10 µM) for 8 days. Thereafter, oil red O staining and its quantification were performed. The results are representative of at least seven different experiments. The quantification results are expressed as mean ± SE (*n* = 8); *** *p* < 0.001. (**C**–**G**) After reaching confluency, the cells were incubated in adipogenic medium with PD98059 (0, 10, and 20 µM) for 4 days. The mRNA expression of adipogenic differentiation markers (*Pparγ*, *C/ebpα*, *Fas*, *Fabp4*, and *Apn*) was examined by real-time PCR. The results are expressed as mean ± SE (*n* = 7); ** *p* < 0.01, *** *p* < 0.001. SP: SP600125.

To examine the effect of phloretin-induced JNK inhibition on adipocyte differentiation in ST2 cells, we used the JNK inhibitor SP600125. Oil Red O staining and its quantification showed that the treatment with SP600125 (5 and 10 μM) significantly increased lipid deposition (Figure 5A,B). Moreover, the mRNA expression of *Pparγ*, *C/ebpα*, *Fas*, *Fabp4*, and *Apn* was significantly increased by 10 μM SP600125 (Figure 5C–G). 

### 2.6. The Effects of p38 MAPK Inhibition on Phloretin-Induced Adipogenesis in ST2 Cells

**Figure 6:**
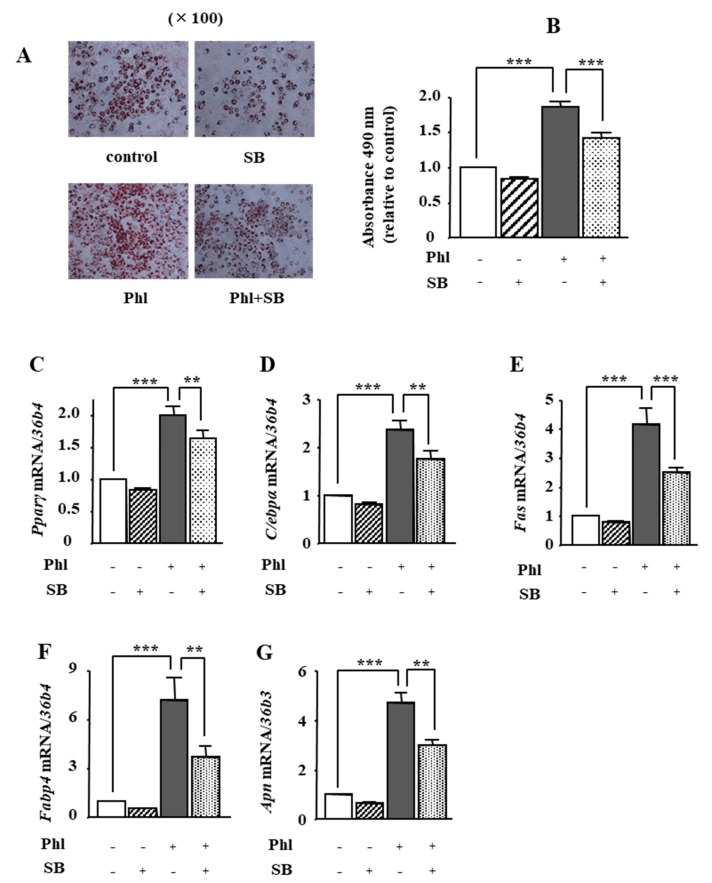
The effects of the p38 MAPK inhibitor SB203580 on phloretin-induced adipogenesis in ST2 cells. (**A**,**B**) After reaching confluency, ST2 cells were incubated in adipogenic medium with phloretin (50 µM) and/or SB203580, a p38 MAPK inhibitor (0, 5, and 10 µM) for 5 days. Thereafter, oil red O staining and its quantification were performed. The results are representative of at least seven different experiments. The quantification results are expressed as mean ± SE (*n* = 6); *** *p* < 0.001. (**C**–**G**) After reaching confluency, the cells were incubated in adipogenic medium with SB203580 (0, 5, and 10 µM) for 4 days. The mRNA expression of adipogenic differentiation markers (*Pparγ*, *C/ebpα*, *Fas*, *Fabp4*, and *Apn*) was examined by real-time PCR. The results are expressed as mean ± SE (*n* = 5); ** *p* < 0.01, *** *p* < 0.0011. Phl: phloretin, SB: SB203580.

We investigated whether phloretin-induced activation of p38 MAPK is involved in adipogenesis in ST2 cells. Oil red O staining and its quantification showed that 50 µM phloretin significantly increased lipid deposition, which was significantly inhibited by co-incubation with the p38 MAPK inhibitor SB203580 (1 µM). In addition, treatment with 50 μM phloretin significantly increased the expression of the adipocyte differentiation markers, *Pparγ*, *C/ebpα*, *Fas*, *Fabp4*, and *Apn*, and co-incubation with 1 μM SB203580 partially but significantly suppressed phloretin-induced upregulation of the adipogenic markers (Figure 6C–G). The treatment with SB203580 alone had no effects on lipid deposition (Figure 6A,B) and adipogenic marker expression (Figure 6C–G).

### 2.7. The Involvement of Glucose Uptake Inhibition on Adipocyte Differentiation in ST2 Cells

**Figure 7:**
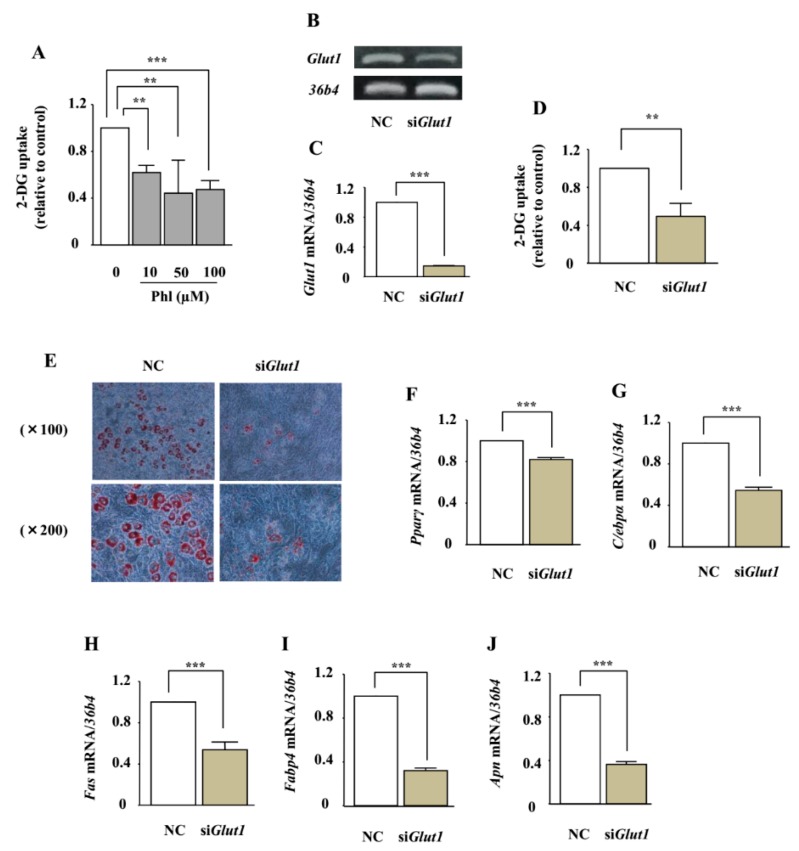
The role of glucose uptake inhibition in adipocyte differentiation in ST2 cells. (**A**) After pre-incubation in Krebs–Ringer Phosphate-Hepes (KRPH) buffer containing 0–100 µM phloretin, the cells were treated with 2-DG containing phloretin 0–100 µM, and then the colorimetric uptake assay was performed. The results are expressed as mean ± SE (*n* ≥ 3); ** *p* < 0.01, *** *p* < 0.001. (**B**,**C**) After transfection with *Glut1* and non-specific control siRNA, the mRNA expression of *Glut1* was examined by RT-PCR (**B**) and real-time PCR (**C**). The housekeeping gene *36b4* was used to normalize the differences in the efficiencies of reverse transcription. The results are expressed as mean ± SE (*n* = 6); *** *p* < 0.001. NC: negative control. (**D**) After transfection with *Glut1* or scramble control siRNA, the cells were incubated in KRPH buffer for 20 min, treated with 2-DG containing 0–100 µM phloretin, and then the colorimetric assay was performed. The results are expressed as mean ± SE (*n* = 6); ** *p* < 0.01. (**E**) After transfection with *Glut1* and non-specific control siRNA, the cells were incubated in adipogenic medium for 8 days, and oil red O staining was performed. (**F**–**J**) After transfection with *Glut1* and non-specific control siRNA, the cells were incubated in adipogenic differentiation medium for 4 days. The mRNA expression of adipogenic differentiation markers (*Pparγ*, *C/ebpα*, *Fas*, *Fabp4*, and *Apn*) was examined by real-time PCR. The results are expressed as mean ± SE (*n* ≥ 4); *** *p* < 0.001.

The effect of phloretin on glucose uptake in ST2 cells was measured by using the 2-deoxyglucose (2-DG) colorimetric assay. Treatment with phloretin (10–100 µM) significantly inhibited 2-DG uptake in the cells (Figure 7A). We have previously shown that ST2 cells express only GLUT1, among all the members of the GLUT subfamily [29]. We investigated whether the adipogenic effects of phloretin are mediated by glucose uptake inhibition induced by *Glut1* silencing. RT-PCR and real-time PCR showed that *Glut1* small interfering RNA (siRNA) significantly suppressed the expression of *Glut1* mRNA, compared with the negative control siRNA (Figure 7B,C), and decreased glucose uptake (Figure 7D). In contrast to the effects of phloretin, oil red O staining showed that the knockdown of *Glut1* markedly suppressed lipid accumulation (Figure 7E). Furthermore, the knockdown of *Glut1* significantly decreased the expression of the adipogenic markers *Pparγ*, *C/ebpα*, *Fas*, *Fabp4*, and *Apn* (Figure 7F–J).

### 2.8. The Effect of Phloretin on Adipocyte Differentiation in Glut1 Knocked-Down ST2 Cells

**Figure 8:**
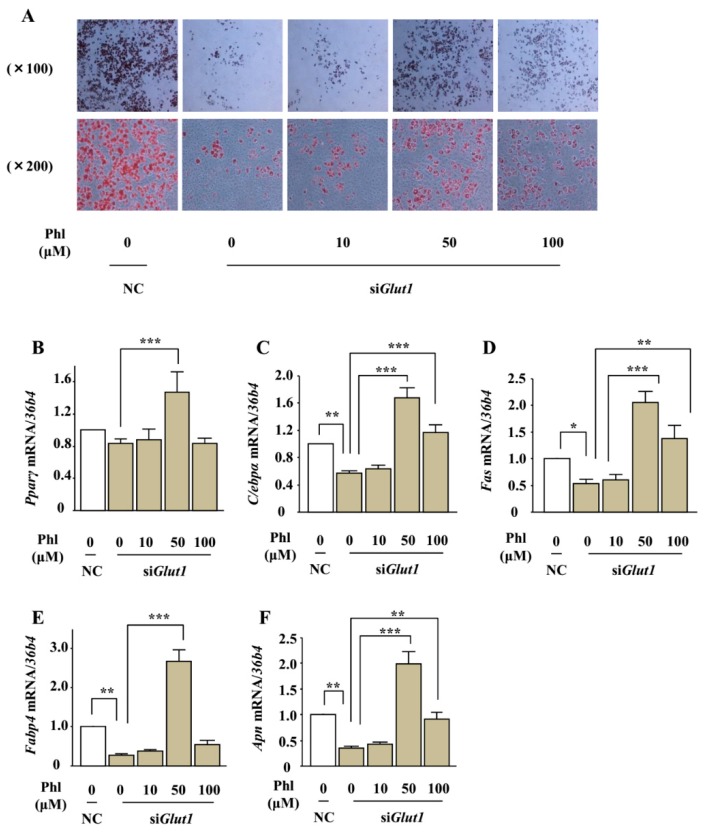
The effect of phloretin on adipocyte differentiation in *Glut1* knocked-down ST2 cells. (**A**) After transfection with control or *Glut1* siRNA, the cells were incubated in adipogenic medium containing phloretin 0–100 μM, and oil red O staining was performed at day 8. (**B**–**F**) After transfection with control or *Glut1* siRNA, the cells were incubated in adipogenic medium containing phloretin 0–100 μM for 4 days. The mRNA expression of adipogenic differentiation markers (*Pparγ*, *C/ebpα*, *Fas*, *Fabp4*, and *Apn*) was examined by real-time PCR. The results are expressed as mean ± SE (*n* ≥ 4); * *p* < 0.05, ** *p* < 0.01, *** *p* < 0.001. NC: negative control, Phl: phloretin.

To further investigate the involvement of glucose uptake inhibition in phloretin-induced adipogenesis, we examined the effects of phloretin on lipid deposition and expression of adipocyte differentiation markers in *Glut1* knocked-down ST2 cells. After transfection with control or *Glut1* siRNA, the cells were incubated in adipogenic medium containing phloretin at a concentration of 0 to 100 μM. Oil red O staining showed that the silencing of *Glut1* decreased lipid deposition; on the other hand, treatment with phloretin 10–100 µM increased lipid deposition even in the *Glut1* knocked-down cells (Figure 8A). In addition, real-time PCR showed that the silencing of *Glut1* significantly decreased the expression of *C/ebpα*, *Fas*, *Fabp4*, and *Apn* and slightly but not significantly decreased the expression of *Pparγ*, that treatment with phloretin 50 μM facilitated the expression of *Pparγ* and *Apn*, and that phloretin 50 and 100 μM enhanced the expression of *C/ebpα*, *Fas*, and *Fabp4* in *Glut1* knocked-down ST2 cells (Figure 8B–F). These findings demonstrated that phloretin promoted adipogenesis in ST2 cells independent of glucose uptake inhibition.

## 3. Discussion

Previous studies have shown that phloretin affects adipocyte differentiation [17,18] and lipid accumulation [19] in preadipocytes. In the present study, for the first time, we showed that phloretin promoted the commitment of MSCs to adipocytes via the suppression of ERK1/2 and JNK signals and the activation of p38 MAPK. Although phloretin enhanced AMPK phosphorylation, AMPK was not found to be involved in phloretin-induced adipogenesis. Phloretin is a known GLUT inhibitor [12]. However, inhibition of glucose uptake induced by *Glut1* silencing did not promote but, rather, suppressed adipogenesis. Moreover, phloretin stimulated adipogenesis even in the *Glut1* knocked-down cells, indicating that phloretin’s adipogenic effect was not mediated by glucose uptake inhibition. 

The involvement of MAPKs in adipogenesis has been reported. However, to our knowledge, there are no studies examining the roles of phloretin–MAPKs signalling in adipocytes so far. It has been reported that ERK signals are required in the stage of mitotic clonal expansion [2,28,32,33], whereas ERK signals suppress adipocyte differentiation in the terminal differentiation phase [2,28,34]. Several studies have shown that the activation of ERK signal suppresses adipocyte differentiation by inhibiting PPARγ activity [35,36,37]. In this study, phloretin suppressed the phosphorylation of ERK, and ERK inhibition increased PPARγ expression and induced adipogenesis. These findings are consistent with those from previous studies. The role of p38 MAPK in adipogenesis is still controversial. While some studies have demonstrated that activation of p38 MAPK promotes adipogenesis [38,39,40], opposite results have also been reported [41,42]. Studies by Engelman et al. and Aouadi et al. suggested that p38 MAPK activity may be required in the initial stage of adipogenesis, and that suppression of p38 MAPK may be necessary in the later stage of adipogenesis [43,44]. Considering these results, the diverse role of p38 MAPK during adipogenesis might be one possible cause of the discrepancy. The present study demonstrated that transient activation of p38 MAPK signalling by phloretin promoted adipogenesis. In addition, inhibition of p38 MAPK by phloretin might be associated with its adipogenic effect in the later stage. With regard to JNK signalling, our data suggest that phloretin enhanced adipogenesis via JNK suppression. Although several studies have reported inconsistent results about the role of JNK signalling in adipogenesis [45,46,47,48,49,50], some studies have reported that inactivation of JNK promotes adipogenesis [49,50], which is consistent with our results. Further studies are necessary to elucidate the roles of JNK signalling in adipogenesis.

AMPK is known to be an intracellular energy sensor and is activated when the intracellular AMP/ATP ratio increases during intracellular energy shortage [22,23]. Therefore, the phloretin-induced AMPK phosphorylation observed in this study may indicate glucose uptake inhibition. It is reported that the pharmacological activation of AMPK suppresses adipogenesis in preadipocytes [25,26]. By contrast, we showed that the AMPK inhibitor ara-A did not inhibit phloretin-induced increase in adipogenic marker expression, indicating that AMPK activation by phloretin did not affect adipogenesis. Moreover, while phloretin treatment decreased glucose uptake in ST2 cells, the inhibition of glucose uptake by *Glut1* silencing did not increase but, rather, suppressed adipogenesis. These findings are consistent with those of Batchvarova et al. and Carlson et al., who showed that glucose deprivation impaired adipogenesis by upregulation of the C/EBP homologous protein, a negative regulator of adipogenesis, in 3T3-L1 preadipocytes [51,52]. Therefore, the adipogenic effect of phloretin on ST2 cells does not seem to be mediated by AMPK activation or glucose uptake inhibition. Further studies are necessary to clarify the roles of phloretin-induced AMPK activation and glucose uptake inhibition.

Adipose tissue regulates whole body energy metabolism by secreting adipokines in addition to storing redundant nutrients [1]. In vitro and in vivo studies by Shu et al. and Shen et al. demonstrated that oral administration of phloretin decreased the plasma glucose levels in diabetic rodent models [18,20]. APN is one of the adipokines that enhance insulin sensitivity [1]. In the present study, phloretin increased *Apn* expression, consistent with previous studies [17,53]. Therefore, phloretin-induced increase of *Apn* expression may also contribute to the improvement of glucose intolerance caused by phloretin. On the other hand, Alsanea et al. reported that phloretin decreased fat mass and improved obesity, insulin resistance, fatty liver, and inflammation in adipose tissues in a high-fat diet-induced obese mouse model [21]. Thus, phloretin may ameliorate metabolic dysregulation via pleiotropic effects, such as an anti-inflammatory effect, other than its effects on adipogenesis. 

In conclusion, our study shows that phloretin enhanced adipogenesis and adiponectin expression by inhibition of ERK1/2 and JNK pathways and activation of p38 MAPK in mouse marrow stromal ST2 cells. Neither glucose uptake inhibition nor AMPK activation were associated with phloretin-induced adipogenic effects. These findings indicate that phloretin may influence energy metabolism by affecting adipogenesis and adiponectin expression. 

## 4. Materials and Methods

### 4.1. Reagents

Cell culture medium and supplements were purchased from GIBCO-BRL (Rockville, MD, USA). Phloretin, the AMPK inhibitor ara-A, the MEK inhibitor PD98059, the JNK inhibitor SP600125, the p38 inhibitor SB203580, and the anti-β actin antibody were purchased from Sigma–Aldrich (St. Louis, MO, USA). Antibodies against phospho-AMPKα (Thr172), total AMPKα, phospho-ERK1/2, total-ERK1/2, phospho-SAPK/JNK, total-SAPK/JNK, phospho-p38α MAPK, and total-p38α MAPK were purchased from Cell Signaling Technology (Beverly, MA, USA). 

### 4.2. Cell Culture

The mouse MSC line ST2 was purchased from the RIKEN Cell Bank (Tsukuba, Japan). The cells were cultured in α-minimum essential medium (α-MEM) supplemented with 10% fetal bovine serum (FBS) and 1% penicillin–streptomycin in 5% CO_2_ at 37 °C. The medium was changed twice a week, and the cells were passaged when they were 80% confluent. For adipogenic differentiation, the cells were incubated in α-MEM supplemented with 10 μg/mL bovine insulin, 0.5 mM 3-isobutyl-1-methylxanthine, and 2.5 µM dexamethasone for 2 days (from day 0 to 2), and then incubated in α-MEM supplemented with 10 μg/mL bovine insulin for 6 days (from day 2 to 8). The medium was changed every 2 days.

### 4.3. Oil Red O Staining

The cells were incubated in adipogenic differentiation media. The cells were washed with phosphate-buffered saline (PBS) twice and fixed with 10% formalin for 10 min. Then, the cells were washed twice with PBS and treated with 60% (*v*/*v*) isopropanol for 1 min. Following this, isopropanol was aspirated, and oil red O staining was carried out with the addition of 0.3% (*w*/*v*) oil red O (Sigma, St. Louis, MO, USA) in isopropanol/water (60:40) for 20 min at room temperature. The oil red O stain was removed, and the cells were washed with 60% (*v*/*v*) isopropanol and again with PBS twice. Then, the cells were analysed under a microscope. For the quantification of lipid deposition, the cells were treated with 100% isopropanol, and the absorbance at 490 nm was measured with a microplate reader. The results are expressed relative to the control. 

### 4.4. Quantification of Gene Expression Using Real-Time Polymerase Chain Reaction (PCR)

Total RNA was extracted from the cultured ST2 cells using TRIzol reagent (Invitrogen, San Diego, CA, USA) according to the manufacturer’s recommended protocol. We used 2 μg total RNA for the synthesis of single-stranded cDNA (cDNA synthesis kit; Invitrogen). Then, we used SYBR green chemistry to examine the mRNA expression of the adipogenic markers *Pparγ*, *C/ebpα*, *Fas*, *Fabp4*, and *Apn*, as well as that of *Glut1*. A housekeeping gene, *36b4*, was used to normalize the differences in the efficiencies of reverse transcription. The primer sequences are listed in Table 1. Real-time PCR was performed with 1 μL of cDNA in a 25 μL reaction volume using the Thermal Cycler Dice Real Time System II (Takara Bio, Shiga, Japan). The double-stranded DNA-specific dye SYBR Green I was incorporated into the PCR buffer provided in the SYBR Green Real-time PCR Master Mix (Toyobo Co. Ltd., Tokyo, Japan) to enable the quantitative detection of the PCR product. The PCR conditions were: 95 °C for 15 min, followed by 40 cycles of denaturation at 94 °C for 15 s, and annealing and extension at 60 °C for 1 min. 

### 4.5. Western Blot Analysis

For western blot analysis, the cells were plated in six-well plates and cultured as described above. After reaching confluency, the cells were treated with each agent. The cells were rinsed with ice-cold PBS and scraped on ice into lysis buffer (Bio-Rad, Hercules, CA, USA) containing 65.8 mM Tris-HCl (pH 6.8), 26.3% (*w*/*v*) glycerol, 2.1% sodium dodecyl sulphate (SDS), and 0.01% bromophenol blue, to which 2-mercaptoethanol was added to achieve a final concentration of 5%. The cell lysates were sonicated for 20 s. The cell lysates were electrophoresed on 10% SDS-polyacrylamide gels and transferred onto a nitrocellulose membrane (Bio-Rad). The blots were blocked with Tris-buffered saline (TBS) containing 1% Tween 20 (Bio-Rad) and 3% bovine serum albumin (BSA) for 1 h at 4 °C. Then, the blots were incubated overnight at 4 °C with gentle shaking with a primary antibody against phosphorylated ERK1/2 (1:2000 dilution) and other primary antibodies (1:1000 dilution). These blots were extensively washed with TBS containing 1% Tween 20 and were further incubated with a 1:5000 dilution of horseradish peroxidase-coupled IgG of specified animal species (rabbit or mouse) matched to the primary antibodies in TBS for 30 min at 4 °C. The blots were then washed, and the signal was visualised using an enhanced chemiluminescence technique.

### 4.6. RNA Interference for GLUT1

RNA interference was used to knock down the expression of GLUT1 in ST2 cells. SMARTpool reagents for GLUT1 and non-specific control siRNA duplexes were designed and synthesised by Dharmacon (Lafayette, CO, USA). For gene knockdown experiments, ST2 cells were seeded in six-well plates and cultured at 37 °C for 48 h in α-MEM containing 10% FBS and antibiotics, followed by 24 h of incubation in medium without antibiotics. The cells were transfected with siRNAs (50 nM) using DharmaFECT 1 transfection reagent (Dharmacon, Lafayette, CO, USA) for 24 h according to the manufacturer’s instructions. Then, the cells were incubated in α-MEM supplemented with 10% FBS and antibiotics for additional 24 h to reach confluency. 

### 4.7. Reverse Transcription PCR (RT-PCR) Analysis to Determine the Expression of GLUT1

To confirm the silencing efficiency of *Glut1* mRNA by siRNA, we performed RT-PCR. After siRNA transfection, total RNA was extracted using TRIzol reagent (Invitrogen, San Diego, CA) according to the manufacturer’s recommended protocol. We used 2 μg total RNA for the synthesis of single-stranded cDNA (cDNA synthesis kit; Invitrogen). The PCR conditions were as follows: denaturation at 94.0 °C for 45 s, annealing at 60.0 °C for 30 s, and elongation at 72 °C for 45 s for 30 cycles. The PCR products were separated by electrophoresis on a 1.8% agarose gel and were visualised using ethidium bromide staining with ultraviolet (UV) light, using the Electronic UV transilluminator (Toyobo Co. Ltd.). 

### 4.8. 2-DG Uptake Colorimetric Assay

To examine the effects of phloretin and *Glut1* silencing on glucose uptake, glucose uptake was examined using a Glucose Uptake Assay Kit (BioVision, Hannover, Germany). The cells were incubated in 96-well plates until they reached confluency. The cells were starved for glucose by incubating in KRPH buffer (20 mM HEPES, 5 mM KH_2_PO_4_, 1 mM MgSO_4_, 1 mM CaCl_2_, 136 mM NaCl, and 4.7 mM KCl, pH 7.4) containing 2% BSA with phloretin (0 to 100 μM) for 20 min. After the buffer was removed, the cells were further incubated with 10 μL of 10 mM 2-DG and phloretin (0 to 100 μM) for 20 min. The Reaction Mix A (total 10 μL: assay buffer 8 μL and enzyme mix 2 μL) was added and the cells were incubated for 60 min. The extraction buffer (90 μL) was added and the cells were incubated at 85.0 °C for 40 min. The plate was cooled on ice for 5 min, and 12 μL neutralization buffer was added. Thereafter, the Reaction Mix B (total 38 μL: glutathione reductase 20 μL, substrate DTNB 16 μL, and recycling mix 2 μL) was added, and the absorbance at 405 nm was measured with a microplate reader. The amount of 2-DG uptake is proportional to the absorbance. The results are expressed relative to the control.

### 4.9. Statistics

The results are expressed as means ± standard error of mean (SEM). Statistical evaluations for differences between groups were performed using one-way analysis of variance (ANOVA) followed by Fisher’s protected least significant difference test. For all statistical tests, a value of *p* < 0.05 was considered statistically significant.

## Figures and Tables

**Table 1:** Primer sequences used for various genes.

Gene Name	Primers (5′-3′)	Accession No.
*36b4*	AAGCGCGTCCTGGCATTGTCT	NM_007475
	CCGCAGGGGCAGCAGTGGT	
*Pparγ*	GTCTGTGGGGATAAAGCATC	NM_001127330.2
	CTGATGGCATTGTGAGACAT	
*C/ebpα*	TGAAGGAACTTGAAGCACA	NM_001287521.1
	TCAGAGCAAAACCAAAACAA	
*Fas*	CCCTTGATGAAGAGGGATCA	NM_007988.3
	ACTCCACAGGTGGGAACAAG	
*Fabp4*	TGGAAAGTCGACCACCATAAA	NM_024406.2
	GTCACGCCTTTCATGACACA	
*Apn*	TGTTGGAATGACAGGAGCTG	NM_009605.5
	TCCTTTTCACAAAGCCACACTAT	
*Glut1*	CGTCGTTGGCATCCTTAT	NM_011400.3
	TTCTTCAGCACACTCTTGG	

## Abbreviations

APN	adiponectin
C/EBP	CCAAT/enhancer-binding protein
PPARγ	peroxisome proliferator-activated receptor γ
GLUT	glucose transporter
MSC	marrow stromal cell
AMPK	adenosine monophosphate-activated protein kinase
MAPK	mitogen-activated protein kinase
ERK	extracellular signal-regulated kinase
JNK	c-Jun N-terminal kinase
MEK	MAPK/ERK kinase
α-MEM	α-minimum essential medium
FBS	fetal bovine serum
PBS	phosphate-buffered saline
PCR	polymerase chain reaction
FAS	fatty acid synthase
FABP4	fatty acid-binding protein 4
SDS	sodium dodecyl sulphate
TBS	Tris-buffered saline
BSA	bovine serum albumin
siRNA	small interfering RNA
UV	ultraviolet
2-DG	2-deoxyglucose
KRPH buffer	Krebs–Ringer Phosphate-Hepes buffer
SEM	standard error of mean
ANOVA	analysis of variance
